# AI-enhanced X-ray diffraction analysis: towards real-time mineral phase identification and quantification

**DOI:** 10.1107/S2052252524008157

**Published:** 2024-08-28

**Authors:** Nikolaos I. Prasianakis

**Affiliations:** a Laboratory for Waste Management Paul Scherrer Institute Forschungsstrasse 111 Villigen PSI 5232Switzerland

**Keywords:** computational modeling, convolutional neural networks, powder X-ray diffraction, mineral phase identification

## Abstract

The use of convolutional neural networks can revolutionize XRD analysis by significantly reducing processing times. Demonstration against synthetic and real mineral mixture data provide a first assessment of the accuracy of such methods.

There are a large number of scientific disciplines which benefit from advanced X-ray-based analytical techniques. The X-ray diffraction (XRD) method is a powerful technique which can provide qualitative and quantitative information about the crystallographic structure and composition of matter in a non-destructive way. Such information is crucial in several fields, such as in materials science for novel materials research, or in environmental and geological sciences where it improves the understanding of subsurface composition and chemistry, which is essential for resource exploitation and pollutant dispersion studies, to name a few.

The XRD techniques range from powder XRD, where the samples are provided in a homogeneous powder form, to XRD computed tomography (XRD-CT) where spatial investigation of heterogeneous samples in 2D and 3D is possible. At the same time, XRD techniques are paired with several synchrotron X-ray-based techniques and provide crucial complementary information (Allen, 2023 ▸). The working principle of XRD is based on the diffraction of X-rays resulting from their interaction with the crystalline planes of the considered material. The diffracted X-rays are collected by a specialized detector, where the diffraction pattern is recorded as a plot of the diffracted X-ray intensity versus the scattering angle. These diffraction patterns are subsequently analysed and compared with reference patterns. Each crystal is characterized by a unique fingerprint of peaks which allows the identification of multiple crystals within the same signal. Signal fitting to known patterns is a complex computationally demanding process, especially when multiple crystals are present. While powder XRD typically provides a single diffraction pattern, in XRD-CT there is one XRD pattern for each pixel within the considered 2D/3D domain resulting in multi-dimensional big data. The number of pixels within such a 3D tomogram can easily become very large (>10^5^) introducing several challenges related to tomographic reconstruction, high-throughput data acquisition and their respective modelling (Hayashi *et al.*, 2015 ▸, 2019 ▸; Finegan *et al.*, 2020 ▸). More specifically, the model refinement computations are, due to the large number of the XRD-CT patterns that have to be processed, a few orders of magnitude slower than powder XRD computations. A potential solution to accelerate these computationally intensive processes is the application of artificial intelligence (AI) techniques.

In recent years, developments in AI and machine learning have been quite impressive opening new avenues in numerical modelling and data interpretation. There are already several applications which take advantage of these advancements, ranging from the acceleration of reactive transport simulations using machine learning (Jatnieks *et al.*, 2016 ▸), to image and pattern recognition in the sense of ultrafast processing and detection which surpasses human capability (Ragone *et al.*, 2023 ▸; Boiger *et al.*, 2024 ▸; Omori *et al.*, 2023 ▸). More specifically, for XRD measurement interpretation, recent seminal works of Dong *et al.* (2021 ▸, 2023 ▸) and Lee *et al.* (2021 ▸) have implemented convolutional neural network (CNN) models to extract important information like phase identification and lattice parameters directly from XRD patterns. In Dong *et al.*, it is demonstrated that using a trained CNN model the results can be interpreted up to three orders of magnitude faster, while Lee *et al.* (2021 ▸) reported achieving the completion of the task in a few seconds instead of several hours, compared with the use of traditional techniques (Rietveld method).

In the same direction, there are the recent efforts for phase quantification using deep neural network processing of XRD patterns (Simonnet *et al.*, 2024 ▸; Poline *et al.*, 2024 ▸). In the article by Simonnet *et al.* (2024 ▸) in this issue of **IUCrJ**, the authors aim to introduce a CNN-based method for direct mineral phase quantification from the XRD signals. The training of these models requires a very large dataset and for that purpose pure XRD samples of four minerals and their mixtures are generated synthetically, using crystallographic information files. Interestingly, the authors incorporate the effect of the instrumental factors into the synthetic dataset, in order to increase the model realism and to accurately account for the exact experimental geometry, such as the wavelength function and the attenuation factor.

This approach results in a significant improvement in the accuracy and efficiency of the model in the considered real mineral mixture example, as shown in Fig. 1 ▸ (Simonnet *et al.*, 2024 ▸). Although the authors provide information, validation benchmarks and examples for a four-component system, the extension to systems composed of more than four minerals, which is common in practice, appears straightforward if the same methodology is followed. However, it should be noted that the number of training samples as well as the training efforts to build a more general model are expected to also increase hand in hand with the number of components, in order to maintain the same levels of accuracy.

It is of paramount importance and an excellent example of open research that the code has been made freely available as open-access software on the GitHub software development platform (GitHub – titouansimonnet/XRD_Proportion_Inference, https://github.com/titouansimonnet/XRD_Proportion_Inference). This repository and version control system includes well-documented input files, the code for generating the training datasets (synthetic XRD patterns), and the machine learning model architecture and parameters, as well as the test cases. This provides to the scientists in the community an excellent starting point to familiarize themselves with the tool, to use it, to optimize it and to extend it for the description of more complex systems.

AI-based tools enable ultrafast interpretation and processing of XRD patterns, significantly reducing the time required compared with traditional methods. The speed of interpretation and the resulting reduction in computational cost should not be underestimated. With these advancements, there is the potential to develop real-time experimental companions capable of interpreting and possibly reducing the dimensionality of the acquired data during the experiment. This opens new horizons such as conducting dynamic experiments with parallel XRD analysis, similar to Cai *et al.* (2020 ▸), and obtaining real-time high-resolution 3D information, which could be further used for the steering and adjustment of the experiment. Additionally, integrating real-time experimental data with highly sophisticated physical modelling algorithms can facilitate the development of digital twins of experiments, supporting the fitting of physical model parameters of interest and the exploration of parametric system responses within a single experiment.

## Figures and Tables

**Figure 1:**
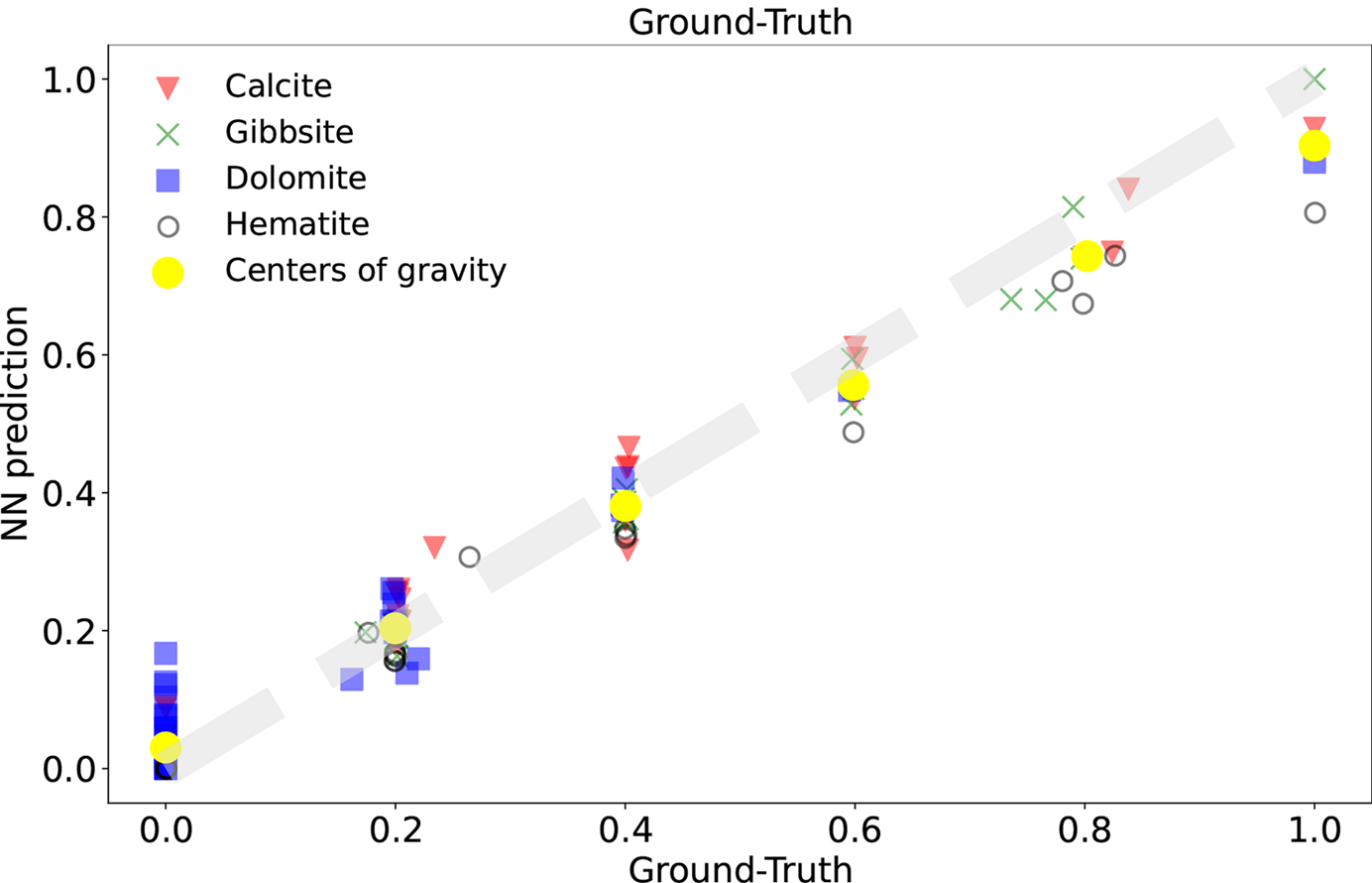
Scatter plot of the CNN model prediction versus the ground truth for mixtures composed of four mineral phases (calcite, gibbsite, dolomite and hematite). The training database takes into account the instrumental effects [DwIE in Simonnet *et al.* (2024 ▸)]. The centers of gravity are the weighted mean of each data points subset. Figure reproduced from Simonnet *et al.* (2024 ▸).
